# Treatment of Philadelphia chromosome-positive acute lymphoblastic leukemia in Jehovah's witness patients

**DOI:** 10.1016/j.lrr.2024.100474

**Published:** 2024-07-24

**Authors:** Jeffrey Baron, Manu R. Pandey, Elizabeth A. Griffiths, Eunice S. Wang

**Affiliations:** a Pharmacy Department, Roswell Park Comprehensive Cancer Center, Buffalo, NY, USA; b Leukemia Service, Department of Medicine, Roswell Park Comprehensive Cancer Center, Buffalo, NY, USA

**Keywords:** Philadelphia chromosome positive acute lymphoblastic leukemia, Jehovah's witness, Tyrosine kinase inhibitors, Low-dose chemotherapy, Growth factors, Supportive care management

Dear Editor,

In caring for individuals with diverse religious beliefs, communication and careful consideration is paramount to ensure that an optimal treatment regimen is pursued congruent with their faith. This is exemplified in the treatment of hematological cancers diagnosed in individuals of the Jehovah's Witness (JW) faith who often will not accept transfusions of various blood products based on interpretations of several verses from the Old and New Testament (*Genesis 9:4; Leviticus 17:10; Deuteronomy 12:23; Acts 15:28, 29; and Leviticus 17:14*) [1]. While most JW will not accept whole blood or major components, the majority accept other medical therapies including fractional components (i.e. cryoprecipitate, clotting factors), recombinant growth factors, and antibodies [2]. Although lowering transfusion thresholds during chemotherapy can be employed, other modifications to clinical practice are needed when treating individuals of the JW faith to avoid severe cytopenias.

Traditionally, therapy for Philadelphia-positive acute lymphoblastic leukemia (Ph+ ALL) consists of BCR-ABL tyrosine kinase inhibitor (TKI) plus intensive multi-agent chemotherapy, which is associated with prolonged myelosuppression and life-threatening cytopenias. Recent development of novel “chemotherapy-free” regimens combining BCR-ABL TKI with steroids and immunotherapy for older Ph+ALL may also be particularly relevant for treatment of individuals adhering to the JW faith [3]. Here we describe our experience with two adults of JW faith diagnosed with Ph+ ALL. Both refused blood products due to religious reasons. Treatment course and responses are provided (Table 1 and Fig. 1).

**Table 1:** Summary of patient clinical course including therapy modifications and clinical responses.

**Patient No.**	**Age**	**Sex**	**Race**	**Ph+**	**CNS disease at diagnosis?**	**Blasts (%) at diagnosis**	**Treatment**	**Time to next therapy**	**Best response (Peripheral blood)**
1	64	F	African American	Yes	No	54 %	Dasatinib 140 mg daily + dexamethasone 18 mg daily(5 days) –> dasatinib140 mg daily, IT MTX 15 mg monthly (6 cycles)	11 months	MMR
							Ponatinib 45 mg daily–> Ponatinib 30 mg daily after CMR→ Ponatinib 45 mg daily after loss of MMR	15 months	CMR
							Ponatinib 45 mg daily+ MTX 20 mg/m2 D1,8,15 and 22, 6-MP 50 mg three times daily D1–28, Prednisone 200 mg daily D1–5, Vincristine 2 mg D1 every 28 days (3 cycles) –> ponatinib 30 mg	11 months	MMR
							Ponatinib 45 mg daily + blinatumomab (4 cycles)–> ponatinib alone	12 months	Positive inconclusive
							Ponatinib 45 mg daily+ blinatumomab (3 cycles given)–> ponatinib 45 mg alone daily	9 months	MMR
							Ponatinib 45 mg + Spinal radiation (T3-T7 2400cGY over 12 Fx, T12-L3 3000cGY over 10Fx and L4-Sacrum 3000cGY over 10 Fx)+ dexamethasone–> ponatinib 45 mg alone	9 months	–
							ponatinib 45 mg daily+ liposomal vincristine 2.25 mg/m2 every 7 days+ prednisone 40 mg daily	4 months	BCR-ABL <1 %
2	53	F	Hispanic	Yes	No	79 %	**Modified CALGB 10701** **Course V** Not given as patient was not a candidate for stem cell transplant or consolidation chemotherapy **Maintenance** IT MTX 15 mg every 28 days (12 cycles)	–	CMR

IT = intrathecal, MTX = methotrexate, 6MP = 6-mercaptopurine, Ara-C = Cytarabine, MMR = Major molecular response, CMR = complete molecular response, Fx = fractions.

**Fig. 1:**
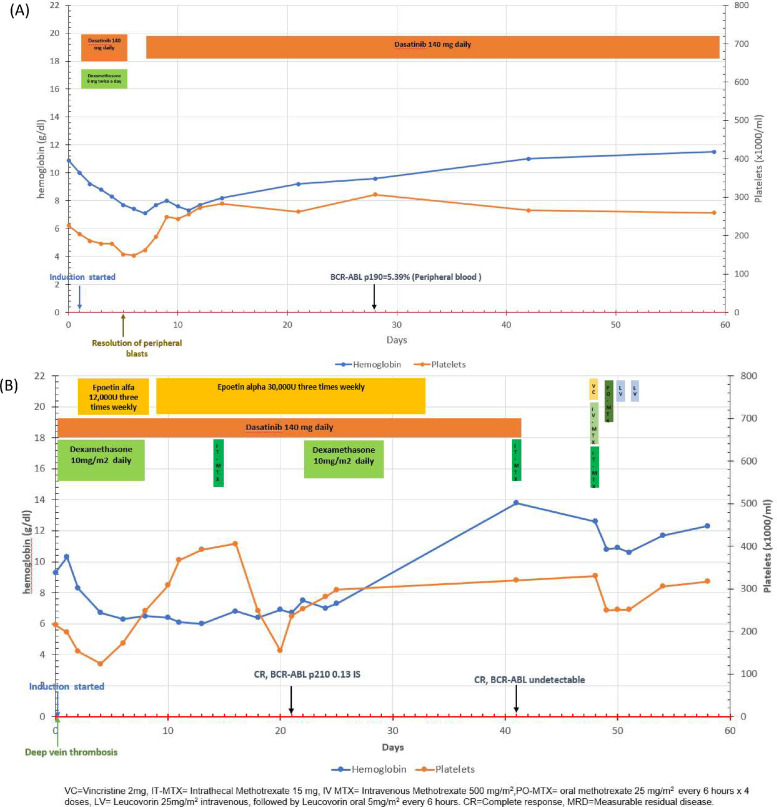
Clinical course including cytopenias, ALL directed therapy, supportive care, and responses in the first 60 days after diagnosis (A) Patient 1, (B) Patient 2.

Patient 1 was a 64-year-old African American woman diagnosed with Ph+ CD20- B-ALL (p190 *BCR-ABL1* transcript 244.61 %). She received therapy with dasatinib and dexamethasone. Hemoglobin (Hgb) reached a nadir of 7.1 g/dl due to occult gastrointestinal bleeding; treated with intravenous pantoprazole without erythropoiesis-stimulating agent (ESA). Post-induction marrow showed a complete morphological and cytogenetic response. Major molecular response (MMR) (*BCR-ABL* transcript ≤0.1 %) was achieved after 3 months of dasatinib. Unfortunately, she experienced MMR loss after eleven months of dasatinib. She started ponatinib 45 mg daily and re-achieved MMR and undetectable *BCR-ABL* transcript (complete molecular response, CMR), after two and six months, respectively. Ponatinib was reduced to 30 mg daily. However, loss of MMR occurred after fourteen months despite dose increase to 45 mg. Therapy was changed to POMP (methotrexate 20 mg/m^2^ on days 1,8,15, 22, 6-mercaptopurine 50 mg TIW, prednisone 200 mg daily on days 1–5, vincristine 2 mg on day 1) plus ponatinib 30 mg daily. She received ESA only with the first cycle and subsequently maintained a Hgb ≥ 7 g/dl without further ESA. Persistent molecular marrow disease (BCR-ABL p190 0.65 %) was noted.

After eleven months, she developed overt relapse. Therapy was changed to blinatumomab+ponatinib 45 mg daily with subsequent undetectable *BCR-ABL* transcript levels. She completed four cycles of blinatumomab+ponatinib followed by ponatinib 45 mg daily. Six months later, loss of MMR was again noted and blinatumomab was re-started. She received 3 cycles, re-obtaining MMR. Nine months later, she presented acutely with symptoms of malignant cord compression; treated with palliative radiation, dexamethasone and continued ponatinib. Four months later, increased peripheral blasts were noted. She started weekly liposomal vincristine (2.25 mg/m^2^) and prednisone in addition to ponatinib with subsequent blast clearance and decline in peripheral *BCR-ABL* transcript. ESA support was needed starting with fourth cycle for worsening anemia with no response. She ultimately passed away due to complications of myocardial infarction from severe anemia and ponatinib therapy.

Patient 2 is a 53-year-old Hispanic woman with Ph+ CD20- B-ALL (p210 *BCR-ABL* 89.94 %). Initiated on steroids and dasatinib induction with ESA support. Bone marrow on day +21 showed complete morphological response and p210 *BCR-ABL* transcript 0.13 %. Bone marrow after course II showed remission with CMR. During course IV, she maintained a Hb > 7 g/dl without ESA. During course V, she received intrathecal methotrexate instead of high dose cytarabine as CNS prophylaxis. She received 16 cycles of POMP maintenance without ESAs with continued CMR. Before cycle 17, patient was admitted with severe anemia (Hgb nadir 4.1 g/dl) and herpes zoster. Bone marrow showed aplasia attributed to concurrent viral illness. Despite supportive measures (ESA, filgrastim & iron supplementation), Hgb worsened with symptomatic chest pain. She received 8 units of polymerized bovine hemoglobin (HBOC-201, Hemopure) until marrow recovery with development of methemoglobinemia, treated with methylene blue and vitamin C. POMP was discontinued. At long-term follow-up almost ten years after diagnosis, she remains disease-free on dasatinib 50 mg daily with continued CMR.

Upfront induction therapy with a BCR-ABL TKI and steroids is an active regimen [4,5]. Such a regimen should be preferred in JW patients as it leads to high rates of remission without the need for cytotoxic therapy. As illustrated here, dasatinib and steroids with ESA resulted in morphologic remission and decreased molecular burden after one cycle consistent with prior report [4]. Similarly, ponatinib with steroids results in >90 % CHR within 6 weeks and 60 % achieving CMR [5]. Ponatinib and blinatumomab results in even more durable responses [6]. However, the potency of ponatinib must be weighed against its cardiovascular risks in patients with severe anemia.

Novel combinations may further negate the need for high-dose consolidative chemotherapy and allogeneic stem cell transplant (allo-SCT) in JW patients. Blinatumomab leads to durable responses in 20 % of patients with low rates of severe cytopenias (<10 %) [7]. In patient 1, we administered blinatumomab and ponatinib as well as low-dose chemotherapy repeatedly with transient disease control across many recurrences. Bloodless auto-SCT has been successfully used in JW patients, resulting in similar overall survival as allo-SCT in ALL patients achieving MMR following auto-SCT [8,9]. Recently, tisagenlecleucel was successfully used in a 20-year-old JW patient with refractory B-ALL, supporting CAR-T therapy as another option [10].

Prevention and treatment of severe anemia for this population is essential. To minimize iatrogenic anemia, pediatric tubes and closed sampling systems are recommended [11]. ESA should be promptly initiated, as it takes approximately two weeks for peak reticulocytosis and nine weeks for maximum Hgb response. Nutritional deficiencies including iron should be adequately repleted and GI prophylaxis should be instituted whenever steroids are used. Luspatercept could also theoretically be used in ESA-nonresponsive JW patients. In patient 2, we used a hemoglobin-based oxygen carrier (HBOC)-201 obtained through an FDA expanded access program to support her until marrow recovery after a viral insult. In a recent series, HBOC-201 successfully salvaged all ten patients with mean Hgb of 3.3 g/dl [12]. However, adverse effects, specifically methemoglobinemia and hypertension, restrict this product's utility to individuals with transient anemia conditions.

Thrombotic and bleeding complications of disseminated intravascular coagulopathy in ALL patients also merit attention, requiring prospective management in JW patients to prevent unforeseen bleeding and blood loss. Drugs that increase bleeding risk should be avoided. Use of oral contraceptives in women of reproductive age as well as prevention of constipation and falls in all patients can further reduce blood loss. Although data for thrombopoietin mimetics in acute leukemias is sparse, these could be considered in JW patients with prolonged severe thrombocytopenia based on expert opinion. Routine anti-coagulation for known thromboses can be safely given with platelets >50,000/mcl.

Recent literature has demonstrated the feasibility of treating other subtypes of acute leukemia in JW patients with chemotherapy, growth factors and targeted agents [[13], [14], [15], [16]]. As illustrated here, we demonstrate that JW patients diagnosed with Ph+ ALL can successfully achieve prolonged overall survival with combinatorial regimens of BCR-ABL TKI, blinatumomab, and low dose chemotherapy supported by liberal growth factor use and hemoglobin alternative.

## Funding

This research did not receive any specific grant from funding agencies in the public, commercial, or not-for-profit sectors.

## CRediT authorship contribution statement

**Jeffrey Baron:** Conceptualization, Writing – original draft, Writing – review & editing, Visualization. **Manu R. Pandey:** Conceptualization, Formal analysis, Investigation, Writing – original draft, Writing – review & editing, Visualization. **Elizabeth A. Griffiths:** Writing – review & editing, Visualization, Supervision. **Eunice S. Wang:** Conceptualization, Writing – original draft, Writing – review & editing, Visualization, Supervision.

## Declaration of competing interest

Jeffrey Baron, none.

Manu R. Pandey, none.

Elizabeth A. Griffiths, Astex Pharmaceuticals – research support, Genentech Inc – research support, Blueprint Medicines Inc – research support, Celldex Therapeutics – research support, Bristol Myers Squibb/Celgene – research support & advisory board, Alexion Pharmaceuticals – research support, Apellis Pharmaceuticals – research support & advisory board, Abbvie Inc – advisory board, Alexion Inc/AstraZeneca Rare Disease – advisory board, CTI Biopharma – advisory board, Novartis – advisory board, Taiho Oncology – advisory board, Takeda Oncology – advisory board, Partner Therapeutics – advisory board, Dresner Foundation – advisory board, AAMDSIF – speaker, Medscape – speaker, ASH – speaker, S Karger Publishing – speaker, MediCom Worldwide – speaker, Physicians Educational Resource – speaker, Picnic Health – advisor, Artis Ventures – advisor, Via Pathways/Elsevier Inc – advisor, NCCN Guidelines – chair & member.

Eunice S. Wang: Advisory board/consulting (Abbvie, Blueprint, Immunogen, Kite, Kura, Qiagen, Rigel, Schrodinger, Stemline, Syndax; Speaker role: Astellas, Pfizer, Dava Data monitoring committees: Abbvie, Gilead; Other: UpToDate.
